# Increasing Role of Oncoplastic Surgery for Breast Cancer

**DOI:** 10.1007/s11912-019-0860-9

**Published:** 2019-12-14

**Authors:** Cary S. Kaufman

**Affiliations:** Department of Surgery, University of Washington, Bellingham Regional Breast Center, 2075 Barkley Blvd., Suite 250, Bellingham, Washington 98226 USA

**Keywords:** Breast surgery, Breast cancer, Oncoplastic surgery, Plastic surgery, Mastectomy, Lumpectomy, Cosmesis, Aesthetic breast cancer, Breast reduction, Ptosis, Aesthetic cancer

## Abstract

**Purpose of Review:**

The goals of surgery for breast cancer have remained the same over the years, to eliminate breast cancer from the breast with the least degree of deformity. With the current expectation of long-term survival after breast cancer treatment, more attention has turned to the cosmetic result of the surgical treatment. Whether lumpectomy or mastectomy, the need for aesthetic improvement was recognized by surgeons both in and outside the USA.

**Recent Findings:**

Oncoplastic surgery combines the skills of the cancer surgeon with those of the plastic surgeon. Sometimes, this means a team approach with a breast surgeon and a plastic surgeon both performing their mutual skills for the patient. Other times, the properly trained breast surgeon may perform some of the plastic techniques at the time of cancer surgery. Breast surgeons are rapidly gaining the ability to improve the post-cancer treatment appearance. To simplify the classification of oncoplastic techniques, we have used lower level, upper level, and highest level. The assignment of techniques to levels is based on both the technique and the surgeon’s training and experience. Much data has accumulated demonstrating the safety and efficacy of the “aesthetic cancer cure.” We describe the development of oncoplastic surgery, the techniques available, matching the right candidate with the right technique, and some comments about the future.

**Summary:**

It is clear from both clinical benefit and patient satisfaction that oncoplastic breast cancer procedures are here to stay. Plastic surgeons will likely focus on the upper- and highest-level procedures while breast/general surgeons will learn lower-level procedures and some of upper-level procedures as needed by their locale. Opportunities to educate breast/general surgeons in these techniques will continue to increase over the next several years. Formal education in oncoplastic surgery during breast fellowships will be necessary to catch up with the rest of the surgical world outside the USA.

## Introduction

Breast cancer surgery has evolved greatly over the years, yet the underlying goals of surgery have remained the same: to eliminate breast cancer from the breast with the least degree of deformity. In the USA each year, there are over 300,000 women diagnosed with breast cancer, and now over 3.5 million survivors live their full lives with the effects of treatment. As long-term survival after breast cancer is commonplace, attention has turned to the cosmetic result of the surgical treatment. Whether provided by a team of a plastic surgeon and a breast surgeon or by a breast surgeon alone, there is increased use of oncoplastic surgery that has improved the overall results of breast cancer care.

## Evolution of Oncoplastic Surgery

In prior years, cancers were larger, margins were not well defined, and radical mastectomy was the standard of care. The next generation found modified radical mastectomy replaced radical mastectomy with equivalent local control and an improvement in appearance.

Initial reconstruction options available after mastectomy were delayed implant reconstruction. Women were encouraged to “live with the mastectomy” so they would appreciate the value of breast reconstruction. Forty years ago, Horton (a plastic surgeon) and Rosato (a general surgeon) reported the use of immediate reconstruction at the time of mastectomy in 17 patients [1]. Yet, immediate breast reconstruction at the time of mastectomy remained an uncommon procedure with limited evidence of oncologic safety [1, 2].

Beyond oncologic safety, there were other reasons why most mastectomy patients did not proceed with any form of reconstruction. The length of time from initial cancer surgery to delayed reconstruction muted patient’s interest in any further surgery. Reimbursement was usually not available since the aesthetic appearance of the breast was considered a cosmetic indulgence. This was prior to the Women’s Health and Cancer Rights Act of 1998 (WHCRA), a federal law that mandates insurance coverage for patients who choose to have breast reconstruction and/or a symmetry procedure after mastectomy [3].

As the implant form of post-mastectomy reconstruction gained in popularity, a national news story changed any momentum that existed. The multiple reports of diseases associated with silicone implant rupture caused many potential patients to avoid any implant reconstruction [4,5,6•]. Women avoided both reconstruction and mastectomy, preferring breast-conserving surgery (BCS) if possible.

Improved screening techniques, earlier diagnosis, better understanding of margins, advocacy by women, and innovative surgeons demonstrated that BCS achieved the dual goals of equivalent local control and survival with less breast deformity. In 1990, a joint statement by the NCI supported the routine use of BCS for early breast cancer [7]. One of the great advantages of BCS was the aesthetic improvement over mastectomy by preserving the breast intact. The aesthetic comparison between a mastectomy and the remaining breast after partial mastectomy was clearly in favor of partial mastectomy. The nipple and most of the original breast remained intact as did the sensation of the breast.

General surgeons applied the Halstedian guidelines of mastectomy to lumpectomy including the concept of an en bloc resection to avoid the risk of spreading cancer. It was considered unsafe to dissect into tissue planes that were not directly related to the excision of the primary cancer. Although cosmesis was an important result, cancer removal and avoidance of local recurrence was paramount. Because the breast was not being removed, it was felt that cosmesis was being achieved and only minimal tunneling to a cancer was accepted to avoid entry into virgin tissue [8].

Yet, 20–30% of BCS patients still had poor cosmetic results [9]. Most breast-conserving surgical procedures (also called partial mastectomy, tylectomy, or lumpectomy) were closed with a simple two-layer closure often with no attempt to close the lumpectomy cavity. If the cavity margins would approximate, they were closed. If not, the lumpectomy cavity was left open and allowed to collect a seroma. Postoperatively, the seroma-filled lumpectomy cavity would maintain a satisfactory appearance for several weeks until radiation treatment. Upon returning from external radiation therapy many weeks later, a significant depression would be found at the site of the seroma. The surgical site would be retracted with poor cosmetic results. The surgeon and radiation therapist would blame each other for the final poor aesthetic appearance and the patient would have to live with the unsatisfactory result.

## Development of Oncoplastic Surgery: an “Aesthetic Cancer Cure”

In the properly chosen patient, both procedures, modified mastectomy and partial mastectomy with whole breast radiation, had equal survival. They both also had issues with their final aesthetic appearance, leaving patients with different versions of deformity. Surgeons felt that they were offering their patients the best cancer survival with cosmesis being of secondary importance. Grateful for their cancer treatment, many patients were too timid to ask for a better aesthetic result. Surgeons were also unaware of other methods to improve cosmesis and underscored the value of a breast cancer cure despite the cosmetic results. Margolese, as well as others, upon reviewing the large National Surgical Adjuvant Breast Project partial mastectomy trials reported that “Many surgeons are of the opinion that an adequate cancer operation cannot be done with cosmetic preservation of the breast” [10, 11]. This was at a time when the average 5-year survival was about 65% and survival was the overriding goal of treatment [12, 13].

With the advent of adjuvant systemic therapies, digital screening mammograms, earlier diagnosis, and women advocates, data demonstrated that long-term survival was common for most women with early breast cancer. These long-term survivors would live with their post-treatment aesthetic results for the rest of their lives. In the footsteps of the original advocate, Rose Kushner, who advocated for the two-step biopsy, avoiding women to wake up with either a normal breast or a radical mastectomy [14], other women advocates identified their post-cancer surgery appearance as an issue to be addressed. They verbalized what impact breast cancer surgery had on their self-image, their relationships, and their lives in general.

In response, several committed surgeons from different countries advanced the proactive notion of an “aesthetic cancer cure.” Among them were Melvin Silverstein, Gail Lebovic, and Scott Spear in the USA; Krishna Clough in France; Werner Audretsch in Germany; and Cicero Urban in Brazil [2, 9, 15–21]. They conceptualized the fusion of cancer excision with the effort to maintain (or improve) the appearance of the breast. They developed and employed techniques to improve aesthetics after both mastectomy and breast-conserving surgery. They promoted adding symmetry procedures on the noncancer breast to complete the aesthetic approach. This concept was originally named “oncoplastic surgery” by Werner Audretsch [16].

One might consider a basic definition of oncoplastic surgery (OPS) as the surgical treatment of breast cancer that combines both oncologic and plastic surgical approaches to breast cancer surgery. But, the functional definition is more dynamic than that. It is a philosophy that breast cancer should be treated surgically by effective cancer surgery while simultaneously maintaining or improving the cosmetic appearance of the breast. That philosophy includes the initial assessment of the patient’s existing anatomy, the patient’s own satisfaction with her own breasts, the expected degree of cancerous tissue removal, and the skillsets necessary to achieve an optimal result. The oncoplastic surgeon evaluates the multiple components of each patient’s clinical presentation and creates a surgical plan to achieve the dual desires of cancer treatment and overall aesthetic appearance. Of key importance is to simultaneously plan both the approach to cosmesis and the oncologic approach before the first incision occurs.

## Working as a Team

Often the breast surgeon may perform lower-level OPS without the aid of a plastic surgeon (Table 1). At other times, based on the breast surgeon’s experience and local community factors, the breast surgeon may team up with a plastic surgeon for other oncoplastic procedures, especially at the time of mastectomy.

**Table 1:** Levels of oncoplastic surgery*

Levels based on the complexity of procedure as well as the degree of surgeon training/experience)	
Lower level	
a. Risk assessment using multidisciplinary model b. Aesthetic principles, evaluation, and techniques c. Comprehensive surgical plan: diagnosis, Rx, adjuvant Rx, follow-up, etc. d. Aesthetic approach to incisions and resection e. Large resections with breast conservation f. Partial breast reconstruction with local tissue flaps/reconstructive lumpectomy g. Accurate marking of the tumor bed for radiation planning and follow-up h. Techniques to recentralize the nipple (crescent, Benelli, etc.) i. Perform skin/nipple-sparing mastectomy j. Perform mastopexy for cancer resection or symmetry	
Upper level**	
a. Perform breast reduction with/without nipple transfer b. Perform augmentation mammoplasty c. Perform mastopexy with implants d. Perform skin/nipple-sparing mastectomy + reconstruction e. Perform reconstruction with expanders/implants f. Perform nipple reconstruction with skin flaps	
Highest level**	
a. Perform implant-based breast reconstruction 1. Retro-pectoral 2. Pre-pectoral b. Perform distant pedicled reconstruction (TRAM flap, latissimus dorsi flap, LICAP flap, etc.) c. Perform distant free flap reconstruction (DIEP flap, etc.)	

*TRAM* transverse rectus abdominis myocutaneous, *LICAP* lateral intercostal artery perforator, *DIEP* deep inferior epigastric perforator

*See Urban et al. [23] and Lebovic [22]

**Often performed by a plastic surgeon in partnership with an OPS-trained breast surgeon

There are many reasons why plastic surgeons may not be available to team up with the breast cancer surgeon. There may be too few plastic surgeons in the geographic area, or the available plastic surgeon may not have an interest in treating breast cancer patients. There also may be inadequate compensation for the plastic surgeon since cancer patients often require more attention than typical reduction mammoplasty patients. Although in many cities there will be a team approach routinely utilized, in resource-limited areas without available plastic surgeons, breast surgeons have learned the skills of these techniques [23–25,26•,^27^,^28^•,^29^,^30^•,^31^].

To achieve optimal results, planning must occur long before the day of surgery. Preoperative planning requires evaluation of multiple factors influencing the surgical approach mentioned above. After the patient is evaluated by both the plastic surgeon and the breast surgeon and after patient approval, the two surgeons will confer and agree on the surgical approach. This includes the incisions to be used and how to approach the portions of the surrounding tissue (nipple or areola preservation, pectoralis muscle and/or fascia, inframammary fold dissection, degree of lymph node dissection, and the contralateral breast). For patients who need radiation postoperatively, some reconstructive methods are best withheld until after radiation while other methods may be used despite radiation. Lesions close to the skin that require the overlying skin to be removed or lesions very close to the nipple will influence the oncoplastic approach. For bilateral procedures or flap procedures, a decision needs to be made whether both surgeons would work at the same time or one surgeon following the other. For all procedures, equipment that aids in lighting, retraction, cautery, and surgical instruments are additional important factors to be addressed.

Beyond these anatomic and oncologic issues, the patient’s wishes are paramount in choosing an oncoplastic approach. Prior to their cancer diagnosis, while some patients are quite happy with their full breast size or ptosis, others have been waiting for an opportunity to have a reduction and/or breast lift. Addressing these unmet needs while excising the cancer broadly improves patient satisfaction.

Although common in Europe and South America [25, 26•, 30•, 32, 33], it is less common for the breast surgeon to be trained in the upper-level techniques (Table 1) in the USA so a team approach is used. For partial mastectomy patients undergoing concurrent reduction, the plastic surgeon will often draw the reduction pattern and direct the location of the incisions on the patient immediately prior to the procedure. Communication is quite important to avoid injury to the blood supply to the nipple of the reconstructed breast. The breast surgeon would then excise the cancer followed by the plastic surgeon performing the reduction and lift. Elsewhere, oncoplastic breast surgeons complete the entire procedure themselves.

## Oncoplastic Surgery Results

One of the previous concerns regarding the addition of oncoplastic surgery at the time of partial mastectomy (lumpectomy) was that cancer may be spread during oncoplastic tissue dissection causing local recurrence and/or metastases. What we have actually seen with current cancer surgery is that the likelihood of local recurrence has gone down [34] progressively over the years for several reasons. The use of adjuvant treatments has become routine (radiation and systemic therapies), and the abilities of imaging to preoperatively localize the extent of cancer has improved (MRI, digital to 3-D mammograms, ultrasounds).

The increased use of OPS has generated data to validate safety and efficacy for tissue rearrangement techniques after partial mastectomy. There are now multiple studies of large numbers of patients to validate OPS results. Carter at M.D. Anderson reported a 5-fold increase in use of OPS for BCS over the years [35••]. They reported no impact on overall survival or recurrence-free survival in their review of over 9000 patients. Similarly, Clough et al. [36, 37•] and others [38–40] found no difference in 5-year survival for patients treated with OPS.

A large meta-analysis by De La Cruz et al. [41••] reviewed 55 studies covering over 6000 patients with a mean follow-up of 50 months which demonstrated “oncologic safety of this procedure in patients with T-1-T2 invasive breast cancer.” They found high rates of overall survival and disease-free survival and low rates of local recurrence, distant recurrence, positive margins, re-excision, conversion to mastectomy, and complications in OPS patients [41••].

Others have reported benefits of OPS such as less frequent positive margins [35••, 36, 39, 41••, 42], fewer re-excisions [41••,^42^–^45^], equal recurrence-free and overall survival [35••, 36, 38, 39, 41••], and excellent patient satisfaction [25, 33, 43, 46]. Concern about complications for oncoplastic procedures during partial mastectomy was shown to be similar or less frequent with oncoplastic procedures [25, 35••, 41••, 46].

One fruitful finding for oncoplastic lumpectomy was the decreased rate of close or positive margins. It is suggested that OPS surgeons may have less frequent close/positive margins since they know they can reconstruct the breast despite widely excising cancers at lumpectomy. Negative margins were found more often after oncoplastic procedures [25, 35••, 42] and acceptably low in other reports [36, 39, 41••]. The ability to close large defects allows the surgeon to be diligent in removing the entire extent of cancer.

## Classification of Oncoplastic Procedures

There have been many efforts to categorize the spectrum of oncoplastic procedures [22, 23, 25, 26•, 30•, 47, 48•]. A principle determining variable is the type of cancer surgery required, mastectomy, or partial mastectomy. Oncoplastic procedures are distinguished by whether the procedure involves volume advancement or volume replacement [48•]. The lumpectomy procedures involve volume advancement while mastectomy involves volume replacement. From that point on, there are OPS methods for each cancer treatment of increasing complexity from simple to complicated [30•].

Collecting the spectrum of procedures relative to the complexity of surgeon performance, we have separated OPS procedures into three levels: lower, upper, and highest levels (Table 1). This practical categorization relates to the surgical training, experience, and abilities of the breast surgeon. The lower-level group represents procedures available to most breast surgeons. Upper levels require increased training when performed by breast surgeons or performed as a team of breast surgeon and plastic surgeon. The highest levels are performed by plastic surgeons (in the USA).

### Lower-Level Oncoplastic Procedures

These procedures are those that involve initial risk assessment of patients, documentation of their baseline anatomy, removal of a moderate amount of breast glandular tissue (about 20–25% of total volume), and performing local advancement post-lumpectomy reconstruction. The procedures are performed by breast surgeons with OPS training that utilize their existing surgical skills. This moderate amount of breast tissue removal can be tolerated by the breast without major tissue manipulation. Once the procedure is planned, the lumpectomy cavity should be closed by advancing local tissue flaps into the cavity. In fuller breast tissue quadrants, such as the upper outer, central, and lower outer, there is usually enough breast tissue to be mobilized into the lumpectomy space. By mobilizing tissue into the lumpectomy cavity, one avoids the retraction and sinking deformity often seen with simple lumpectomy closure. Mobilization of tissue maintains the normal contour of the breast and enhances appearance [49–51]. Almost 40 years ago, Margolese [11] described methods of improving cosmesis at the time of partial mastectomy based on the NSABP experience of 400 patients. His methods of rearranging local tissues into the lumpectomy space are techniques still used today.

When using lower-level procedures, it is necessary to consider where the nipple will be located at the completion of the surgical procedure. Removing tissue from one quadrant will often deviate the nipple towards that quadrant. Knowing how to anatomically mark the breast using the Wise pattern markings is an important skill to identify the ideal location of the nipple. Whether you advance the nipple with these lower-level procedures or not, knowing where it should be is valuable during the operation. If there is excess skin or the nipple needs adjustment, there are OPS procedures for each quadrant. Some procedures available are the crescent mastopexy, the round block or Benelli mastopexy, vertical mastopexy, and inverted T (or anchor) mastopexy [28•, 30•, 32, 52••, 53].

One issue with tissue advancement into the lumpectomy space involves difficulty in targeting radiation to the original lumpectomy cavity margins due to mobilization from their original position [31]. Radiation used for the boost or for external partial breast irradiation [54] is typically targeted to the residual seroma and clips left behind by the surgeon. Yet with increasing degrees of oncoplastic rearrangement, identifying the true lumpectomy cavity margins from tissue mobilization is difficult.

To address this issue, a 3-D bioabsorbable targeting marker [31, 49, 51, 55] has been developed that is sewn to the original lumpectomy cavity which marks the lumpectomy cavity despite surrounding seroma and clips used for hemostasis. The marker is a bioabsorbable spiral sphere with six evenly spaced small titanium clips which may be visualized and targeted from any direction for boost radiation treatment or abbreviated partial breast irradiation (APBI). It provides the radiation oncologist the ability to avoid treatment of oncoplastic dissection planes, tunneling, or other aspects of reconstruction unrelated to the cancer site. The 3-D target dissolves after 12–18 months (although clips are permanent). The combination of oncoplastic reconstruction and 3-D tissue marker has been reported to maintain the contour of the breast as measured by serial mammograms with a mean follow-up of 34 months [50].

In addition to the lower-level procedures removing 20–25% of the breast volume, contralateral symmetry is often an issue in these resections. Most cancer surgeons do not typically consider approaching the contralateral side for symmetry. They often refer the patient to the plastic surgeon with the thought that the cosmetic issue is not in their realm. Yet at one time or another, all breast cancer surgeons have removed the contralateral breast for unilateral cancer when the patient requests it. Contralateral mastectomy has increased in frequency over the last several years [56] and is very often a cosmetic choice rather than a requirement of cancer treatment. If a breast cancer surgeon may perform a mastectomy on the contralateral side for cosmetic reasons, it seems reasonable that a less-than-mastectomy symmetry procedure would be within the scope of a breast cancer surgeon. This rationale has been utilized by many surgeons across the country and in Europe who performed symmetry procedures routinely in the appropriate patients [28•, 30•, 32, 52••, 53].

### Upper-Level Oncoplastic Procedures

#### Volume Advancement

Upper-level volume advancement procedures involve larger amounts of breast tissue (> 25%) to be removed relative to the existing breast. By removing this much tissue, either a mastopexy, a reduction mammoplasty, or both are performed at the same time. Working as a team or individually, one reorients the skin, nipple, and the parenchyma for these procedures. After removing the tumor with surrounding normal breast tissue, one group of these procedures reduces the existing breast and reconstructs the breast while moving the nipple on a vascularized pedicle to a new ideal location of the smaller normal-shaped breast.

#### Volume Replacement

When the patient requires a mastectomy as their cancer operation, the true benefit of oncoplastic surgery comes alive. Although delayed reconstruction has been available for many years, the use of immediate breast reconstruction at the time of mastectomy fits within the definition of oncoplastic surgery. Immediate reconstruction is preferable to delayed reconstruction for several reasons. The tissue is unscarred and easier to work with, symmetry procedures can be done at the same time, it is cost effective to have both the mastectomy and first-stage reconstruction done at the same time, and patient satisfaction is improved. It is at the planning stages of mastectomy that the OPS decisions become paramount. The surgeon needs to assess each of the individual patient parameters to decide which type of volume replacement procedure would be best for this patient. There are many upper-level techniques available with descriptions of various details of the procedures [17, 25, 28•, 30•, 32, 33, 43, 47, 48•, 52••, 57, 58].

### Highest Levels of Volume Replacement (Specialized Training)

These last procedures, free flaps, are performed by specialized centers who have experience and facilities to monitor these patients in the immediate postoperative period. These procedures are beyond the scope and training of the breast or general surgeon. They include free flap procedures such as the deep inferior epigastric perforator (DIEP) flap, superior gluteal artery perforator (SGAP) flap, free transverse rectus abdominis muscle (TRAM) flap, and other free flaps. These take special training and facility resources that limit widespread distribution of these flap procedures. Success of microvascular procedures requires a larger team and is often a referred service. At the same time, some of the most impressive reconstruction results are seen when these flaps are utilized properly in the right hands.

## The Five “S” Variables of Oncoplastic Surgery Patient Assessment: Site/Size/Skin/Shape/Symmetry

In planning an oncoplastic approach, it is useful to identify the five aesthetic components of the breast that OPS evaluates or modifies to achieve an optimal aesthetic result at cancer surgery. One may not need to adjust all five components, but an assessment of all components is needed for each patient. Those five components are (1) the *site* and extent of cancer to be removed, (2) the *size* and volume of the natural breast, (3) the presence and degree of excessive *skin* (ptosis), (4) the desired location of the nipple to create normal *shape* after cancer surgery, and (5) the evaluation of contralateral breast for *symmetry*.

The site or extent of the cancer within the breast is the first data point necessary. The site of cancer may be the area of a lump, an extent of calcifications, or the entire breast. Once the cancer site and the expected volume of tissue to be removed is identified, the remaining four components are evaluated.

The size or volume of the natural breast is a basic underlying variable that impacts all other variables. The two main oncologic procedures will deal with volume differently. The aesthetic improvement after mastectomy requires a volume *replacement* oncoplastic procedure, while after partial mastectomy, an aesthetic *rearrangement* of remaining tissue is necessary. Rearrangement options were discussed earlier.

For the patient who requires a mastectomy with replacement procedures, their individual opinion of the size of her breast is prescriptive for the surgeons. If the patient with a DD cup wishes to be a full C cup, her wishes will direct the choices of reconstruction. Likewise, if she wishes to remain unchanged in size, that also impacts choice of reconstruction.

The most common volume replacement OPS procedure is an implant expander followed by a permanent implant placement. That two-stage process is inconvenient for some patients, and a one-stage biologic wrapped permanent implant placed anterior to the muscle has gained increased use [59–61]. These procedures do not incur the added surgical time of flap preparation or the risks of flap necrosis postoperatively.

Volume replacement procedures ranked by increasing complexity include implant reconstruction (saline or silicone) and minor or major pedicle flap reconstructions such as a TRAM flap. The less complex procedures are performed the most often [62•].

Excessive skin is usually demonstrated by the degree of ptosis noted in the standing position [63]. Patient satisfaction with the existing appearance of the breasts is an important preoperative data point. Preoperative measurement and photographs are necessary to evaluate the final results since both patient and surgeon may have difficulty remembering the preoperative status of the patient.

The shape of the breast is impacted by the location of the nipple. If the patient has minimal ptosis, it is often possible to perform a nipple-sparing mastectomy. The breast surgeon must have experience in dissection behind the nipple to maintain blood supply to the nipple. When viable, the aesthetic results are remarkable after adequate volume replacement with any of the variety of volume replacement methods [64,65•,^66^]. Further research into nipple-sparing mastectomy demonstrated the safety and consistency of this procedure in sites across the country [64,65•,^66^].

When a large resection is to be done in the large ptotic breast, the markings of the breast are very important to choose the proper site of the nipple. Poor planning or improper measurements may defeat an otherwise ideal OPS if the nipple has been moved to an improper location.

When the surgery requires nipple removal, there are several techniques available to create a nipple that are usually performed by the plastic surgeon. In addition, the advent of 3-D nipple tattoos has enhanced the options of nipple reconstruction without a permanently erect nipple (https://www.breastcancer.org/treatment/surgery/reconstruction/types/nipple/3d-nipple-tattoo).

Once the final plan for the ipsilateral side has been completed, any necessary symmetry procedure may be considered for the contralateral breast either simultaneously or at a later date. Patients generally prefer symmetry procedures to occur simultaneously with the cancer surgery, if possible.

A variety of other variables to be considered includes patient characteristics, the patient age, their overall health and comorbidities, their personal desires and satisfaction with their existing anatomy, the importance of their breasts in their appearance and their sexual relationships, prior surgery or scars, type of cancers, need for systemic or radiation treatments, ease of imaging follow-up, and similar issues. Each patient will have an individualized oncoplastic approach that is unique for their situation.

## Learning Oncoplastic Surgery After Residency

One reason why it has taken so long for surgeons to catch on to the oncoplastic approach to breast cancer surgery is that most general surgeons (in contrast to breast focused surgeons) performed only a few breast cancer cases per year [67]. Those surgeons may not have the opportunity to delve deeply into the current state of oncoplastic procedures to enhance aesthetic results. Similarly, many surgical residencies have not had oncoplastic procedure training until recently, and learning these techniques require attending other courses.

In the past, short clerkships were used to learn techniques from the few individuals performing these procedures. Now, there are several courses available both in the USA and abroad due to increasing surgeon interest [68–74]. Hands-on experience is essential in any course one might attend.

Training for oncoplastic surgery is a journey not a single event. It often may take several courses to integrate these techniques into practice. Taking a single course which gives an overabundance of information and expecting to apply all the new data to one’s practice is unreasonable. These techniques are incorporated in practice gradually one at a time. Confidence in performing individual techniques is built over time. Most surgeons attend these educational courses more than once before they master the basic techniques. One slowly incorporates the concepts, the techniques, the approach, and the ability to assess patients into one’s expertise.

## Competence in Oncoplastic Surgery

Demonstrating competence in oncoplastic procedures is a challenge that is both academic and political. Several methods are being tried at this time to validate OPS competence. Surgical privileges are unique products of local/regional environments and traditions, so generalizations are hard to make. With that in mind, a few comments are worth mentioning.

For lower-level procedures, a qualified breast/general surgeon has used all the necessary surgical techniques in prior patients, just not used in the OPS fashion (perhaps with the exception of de-epithelization). Those known techniques are applied differently for a different outcome and result. Using these lower-level techniques repeatedly will allow the surgeon to gain confidence. Thereafter, they may gradually incorporate other techniques as time goes forward. Experienced breast surgeons should not need special privileges for most of the lower-level techniques.

Upper-level techniques may be approached as a team with a plastic surgeon. As time and confidence builds, the breast surgeon may expand their oncoplastic array of procedures on their own. Additionally, if the breast surgeon has a reliable plastic surgeon colleague, the team approach can be very successful by itself.

Likewise, symmetry procedures also utilize the same techniques that surgeons use on the breast with cancer. Surgical privileges are based on a combination of education, training, existing skill sets, and observed experience as well as the patient’s diagnosis. Even if there is not a breast cancer on the contralateral breast, many techniques of breast surgery remain very similar. The surgical techniques and oncoplastic goals are quite similar when used on the contralateral breast. The components to qualify for privileges include the evidence that the clinical assessment, past experience, and technical skills are present to perform the desired oncoplastic breast surgery.

## Conclusion

It is clear from both clinical benefit and patient satisfaction that oncoplastic breast cancer procedures are safe and effective and are here to stay. Efforts at training breast/general surgeons in these techniques will continue to increase over the next several years. Plastic surgeons will likely focus on the upper- and highest-level procedures, but breast/general surgeons will learn lower-level procedures and some of upper-level procedures as needed by their patients. Formal education in oncoplastic surgery during breast fellowships will be necessary to catch up with the rest of the surgical world outside the USA.

The future for oncoplastic surgery is here. It is the responsibility and the opportunity for breast/general surgeons to stay on the frontline of breast cancer care and adopt those techniques they feel are appropriate for their own skills and the needs of their patients. That is the best way for surgeons to provide excellent comprehensive breast care to their patients.
